# Dental Colleges

**Published:** 1860-05

**Authors:** 


DENTAL COLLEGES.—At the Annual Commencement of the Pennsylvania College of Dental Surgery, the degree of "Doctor of Dental Surgery" was conferred upon twenty-one candidates. The number of students in attendance at the Baltimore College of Dental Surgery during the past session was seventy, and of these forty-nine were graduated.

